# FT-IR Analysis of Urinary Stones: A Helpful Tool for Clinician Comparison with the Chemical Spot Test

**DOI:** 10.1155/2014/176165

**Published:** 2014-04-27

**Authors:** Aniello Primiano, Silvia Persichilli, Giovanni Gambaro, Pietro Manuel Ferraro, Alessandro D'Addessi, Andrea Cocci, Arcangelo Schiattarella, Cecilia Zuppi, Jacopo Gervasoni

**Affiliations:** ^1^Istituto di Biochimica e Biochimica Clinica, Università Cattolica del Sacro Cuore, Largo Agostino Gemelli 8, 00168 Rome, Italy; ^2^Divisione di Nefrologia Complesso Integrato Columbus, Dipartimento di Scienze Mediche, Policlinico Gemelli Università Cattolica del Sacro Cuore, Largo Agostino Gemelli 8, 00168 Rome, Italy; ^3^Clinica Urologica, Policlinico Gemelli Università Cattolica del Sacro Cuore, Largo Agostino Gemelli 8, 00168 Rome, Italy

## Abstract

*Background.* Kidney stones are a common illness with multifactorial etiopathogenesis. The determination of crystalline and molecular composition and the quantification of all stone components are important to establish the etiology of stones disease but it is often laborious to obtain using the chemical method. The aim of this paper is to compare chemical spot test with FT-IR spectroscopy, for a possible introduction in our laboratory. *Methods.* We analyzed 48 calculi using Urinary Calculi Analysis kit in accordance with the manufacturer's instructions. The same samples were analyzed by FT-IR using the Perkin Elmer Spectrum One FT-IR Spectrometer. All FT-IR spectra of kidney stones were then computer matched against a library of spectra to generate a report on the various components. *Results.* On the basis of FT-IR analysis, the 48 calculi were divided into three groups: pure stone, mixed stone, and pure stone with substances in trace. Results of each group were compared with those obtained with chemical spot test. A general disagreement between methods was observed. *Conclusions.* According to our data, the introduction of the FT-IR technique in clinical chemistry laboratory may be more responsive to clinician expectations.

## 1. Introduction

Nephrolithiasis is a common disease, occurring in both industrialized and developing countries and mainly affecting adults aged 20–60 years [1].

A recent survey in Italy has shown a prevalence of 7.5% in an urban population [2]. Stone formation is the end result of a multistep process in which the balance of factors that promote crystallization of urinary salts and factors that inhibit crystallization is perturbed. Urinary stones may be composed of calcium oxalate monohydrate (COM, whewellite), calcium oxalate dihydrate (COD, weddellite), carbonate apatite (CA, dahllite), ammonium urate, magnesium ammonium phosphate (PAM, Struvite), calcium hydrogen phosphate dihydrate (brushite), uric acid (AU0 anhydrous form and AU2 dihydrate form, uricite) and its salts, cystine, xanthine, 2,8-dihydroxyadenine, and drugs [3].

It is worldwide underlined that the determination of crystalline and molecular composition and the quantification of all stone components are helpful to establish the etiology of stones disease. Different methodologies exist for the analysis of renal stones. These include qualitative “dry” chemical spot tests and quantitative X-ray crystallography, infrared spectroscopy (FT-IR), and “wet” chemistry tests [4].

Chemical spot tests are relatively inaccurate because of false-positive and false-negative results and do not allow distinguishing between the crystalline phases. Among physical methods, X-ray diffraction is appropriate for quantification of mineral samples, but it cannot adequately detect amorphous species such as carbapatite or struvite. FT-IR spectroscopy is the most appropriate technique for stones analysis and is becoming the gold standard for stone analysis [3, 5]. The infrared spectrum originates from the vibrational motion of the molecules. The vibrational frequencies are a kind of fingerprint of the compounds. This property is used for characterization of organic and inorganic compounds present in renal calculi. The band intensities are proportional to the compound concentration and hence qualitative estimations are also obtained. FT-IR spectroscopy leads to unambiguous information about the stone composition, both for main substances and trace elements, all essentials to guide therapy [6].

The aim of this paper is to compare a semiquantitative method (DiaSys) with a quantitative method (FT-IR spectroscopy technique) for urinary stone analysis, in order to introduce in our laboratory a more reliable technique.

## 2. Material and Methods

We analyzed 48 urinary stones, from 48 patients (28 men and 20 women, age range 21–75) from our Divisions of Nephrology and Urology. Stones were analyzed by both spot test and FT-IR method as described below. Shape, colour, size and weight were registered for each stone at the time of delivery. The stones submitted to analysis were washed with deionized water and dried at room temperature for 24 h; subsequently the stones were powdered in a mortar and aliquoted in 2 vials before being subjected to the following analysis.

### 2.1. Chemical Spot Test

Spot test analysis for the qualitative tests of urinary calculi composition was performed according to kit instructions (Urinary Calculi Analysis kit, DiaSys, Diagnostic System GmbH, Holzheim, Germany). This method allows detecting the presence of cystine and following ions usually present in urinary calculi: carbonate, calcium, oxalate, ammonium, phosphate, magnesium, and urate. The assay consists of the addition of chemical reagents labeled R1 to R15 dropwise to the finely pulverized sample and placed into a vessel with 50 mL of distilled water. Then the appearance of certain colors, precipitates, or air bubbles would indicate positive results for one of the ions and cystine [7]. For example, for the phosphate, while shaking, five drops of reagent 9 (ammonium molybdate solution) and five drops of reagent 10 (4-methyl-aminophenol sulfate, sodium bisulfite) were added in reaction vessel; after five-minute incubation the newly appearing colour was matched with the kit colour scale for the semiquantitative analysis.

### 2.2. FT-IR Analysis

The second aliquot of the pulverized stone was mixed with an inert powdered support (dried potassium bromide) in a proportion of 0.5 to 2% in agate mortar. This mixture was transferred into an appropriate die and pressed at 10 t/cm^2^ to form a transparent pellet 13 mm in diameter. The pellet assembled in a holder was placed in the IR beam of the spectrometer. The spectral region investigated was from 4000 to 400 cm^−1^; 32 scans were averaged with a 4 cm^−1^ resolution for each spectrum. A background spectrum was collected before every analysis, for the sample blank.

Again a background spectrum was measured to provide a relative scale for the absorption intensity. Background spectra were performed at air or pure KBr pellet. Spectra were recorded by means of a Perkin Elmer Spectrum One [8].

Spectra were then computer-matched with the Euclidean search application, a tool of SPECTRA NICODOM IR Library (obtained from Nicodom s.r.o., Hlavni 2727 CZ-14100 Praha 4, Czech Republic, EU) that compares the unknown spectrum with reference spectra contained in the library between 4000 and 400 cm^−1^. A report is then generated for the various stone components. The results of the automatic comparison for a spectrum identification were provided as a list of the best-fitting spectra with their score. The score value can range from 0.000 to 1.000. Score 1.000 indicates a perfect likeness between the unknown spectrum and the reference one. In each case, a visual inspection of the spectra was performed to check the results.

### 2.3. Method Comparison

On the basis of FT-IR analysis, the 48 calculi were divided into three groups: pure stone (*n* = 23), mixed stone (*n* = 19), and pure stone with substances in trace (*n* = 6). Results of each group were compared with those obtained with chemical spot test.

Classification criteria were established by comparing results obtained with the two methods in reference to the identification of cystine and the ionic species as follows:

- agreement: when FT-IR and the chemical spot test identify the same components,
- partial agreement: when the chemical spot test identifies the main component detected by FT-IR in addition to other ions not attributable to a particular crystalline species,
- disagreement: when the spot test does not identify the main substance or when it identifies the main component but also other ions referable to particular crystalline species which were not detected by FT-IR.

## 3. Results

The percentage values of major constituents for the chemical spot test and FT-IR method are shown in Tables 1(a) and 1(b), respectively. As clearly shown the major components detected by both techniques were calcium and oxalate. However, the two tests differ remarkably in the detection of oxalate, magnesium, ammonium, and cystine (a higher detection yield by the chemical spot test) and in the detection of urate and carbonate (a lower detection yield by the chemical spot test) (Table 2).

In the pure stone group (Table 3) a partial disagreement between the two methods was shown. In fact, they were in agreement only in 11/23 cases (47.8%), in partial agreement in 4/23 (17.4%), and in disagreement in 8/23 (34.8%) cases.

Tables 4 and 5 show that also results relative to mixed stone and pure stone with substances in trace groups were characterized by a general disagreement. In fact for the mixed stone group the two methods were in agreement only in 3/19 (15.8%), in partial agreement in 4/19 (21.0%), and in disagreement in 12/19 (63.2%) cases.

For the pure stone with substances in trace group the two methods were in agreement in none (0%), in partial agreement in 5/6 (83.3%), and in disagreement in 1/6 (16.7%) cases.

Thus, the mixed stone groups show the major disagreement.

## 4. Discussion

Urolithiasis is a frequent disease whose incidence is progressively increased in the last years in both men and women.

Urinary stone composition is important both for correct diagnosis and for patient follow-up. Among the methods available for urinary stone analysis, chemical analysis has been traditionally used most widely due to its ease and low cost even if this technique is time consuming and necessitates large stone samples.

Guidelines on Urolithiasis of European Association of Urology 2013 underlines the obsolescence of chemical analysis and recommends the use of FT-IR for urinary stone analysis [3]. As reported by some authors, chemical methods presented an error rate from 6.5 to 94% confirming the dramatic inaccuracy of these methods [6]. The reason for such an inertia in the implementation in many laboratories of the FT-IR method while continuing to use qualitative chemical tests most likely resides in the uncertainty on whether an accurate characterization of the stone composition is really useful for the metaphylaxis of nephrolithiasis [9]. No doubt that in very rare forms whose recognition can be only obtained with the FT-IR method (drugs or xanthine or 2,8-dihydroxyadenine stones) this is crucial for the rational treatment of the patient. Actually in this study the spot test could not detect the atazanavir in a stone which was incorrectly recognized as composed by oxalate, a wrong diagnosis which could have led to an incorrect medical treatment. However, these are really very rare cases. Hence, especially for the calcium containing stones still some believe that stone analysis could not be useful in the investigation of renal stone patients [9].

Yet, we think that there is one stronger reason to discourage the use of chemical spot tests, that is, the many relevant drawbacks of these techniques even for the very frequent forms of nephrolithiasis.

In this study, 48 urinary stones were analyzed using both the chemical spot test and the FT-IR method and results were compared. As expected, the most common components detected by both methods were calcium and oxalate. However we observed oxalate in 75.0% and calcium in 77.1% using the FT-IR method, while we observed oxalate in 93,7% and calcium in 81.2% using the chemical spot test. The difference between the two methods in detecting oxalate is remarkable since it was incorrectly recognized in 25.0% more stones.

**Figure 1:**
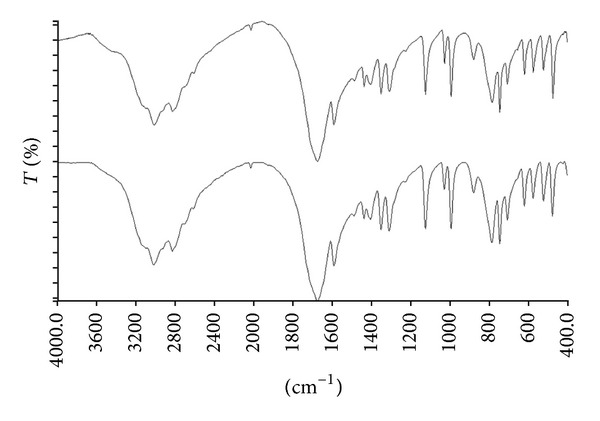
FT-IR spectrum of a uric acid anhydrous stone (top) matched with reference spectra contained in the NICODOM library. The reference spectrum of uric acid anhydrous stone with the best fit value was showed in the bottom.

However, the insufficient discrimination by the chemical spot method of uric acid stones is even more amazing. In fact, the chemical method does not or, only partially, recognize the presence of uric acid in stone samples (number 6) detected by FT-IR analysis (Figure 1). Since uric acid stones represent a significant percentage of urinary stones and deserve a specific medical treatment, a correct diagnosis of uric acid stones is crucial [10].

Also critical is the number of false positive ammonium or magnesium containing stones by the chemical spot method. In fact this finding may suggest to clinicians (despite absence of phosphates) that the stone is constituted by PAM, which mistakenly would lead to the recognition of an infective pathogenesis. Similarly, with this method five cystine containing stones were detected while the true number was two; this finding would lead to specific diagnostic and therapeutic measures which may even worsen the renal stone disease.

Another example can be found in calcium phosphates, which constitute a very heterogeneous group with multiple etiology including infections (in the case of carbonated calcium phosphate and whitlockite), hypercalciuric mechanism (in the case of brushite and octacalcium phosphate), and disorders related to tubular acidification function (in the case of carbonate apatite).

Moreover, there are significant differences on identification of substances present in trace in mixed stones, in fact FT-IR technique shows a high sensitivity and allows an accurate identification of stone composition.

The elaborated treatment of samples and the subjective interpretation of results are the major disadvantages of the spot test, adding a variability out of control to structural method limits. Chemical methods have repeatedly proved to be unreliable in numerous quality control programs, with error rates in identifying certain components above 90% [11]. On the contrast the FT-IR does not show a significant variability because it provides an easier and more standardized sample preparation, and the spectrum interpretation is based on strong scientific principles. The interpretation of results is aided from the use of Nicodom library which however is not always sufficiently sensitive and specific to differentiate species with similar spectral pattern and to detect minor components. Therefore, a skilled operator interpretation remains necessary.

For these reasons, according to these results, the introduction of the FT-IR technique in our clinical laboratory may be more responsive to clinicians' expectations.

## Figures and Tables

**Table:** (a) Chemical spot test analysis

Components detected	Number	Frequency (%)
Oxalate	45/48	93.7
Calcium	39/48	81.2
Magnesium	16/48	33.3
Phosphate	14/48	29.1
Urate	9/48	18.7
Ammonium	7/48	14.5
Cystine	5/48	10.4
Carbonate	3/48	6.2

**Table:** (b) FT-IR analysis

Components detected	Number	Frequency (%)
Calcium oxalate monohydrate	32/48	66.6
Carbonate apatite	16/48	33.3
Anhydrous uric acid	12/48	25.0
Calcium oxalate dihydrate	8/48	16.6
Dihydrate uric acid	2/48	4.1
Magnesium ammonium phosphate	2/48	4.1
Cystine	2/48	4.1
Atazanavir	1/48	1.0

**Table 2:** Comparison between urinary stone composition (frequency %) obtained with chemical spot test (extrapolated associating the single chemical constituents) and FT-IR analysis.

Chemical spot test versus FT-IR in components identification		
Components detected	Frequency (%) by chemical spot test	Frequency (%) by FT-IR
Oxalate	93.7	75.0
Calcium	81.2	77.1
Magnesium	33.3	4.1
Phosphate	29.1	37.5
Urate	18.7	25.0
Ammonium	14.5	4.1
Cystine	10.4	4.1
Carbonate	6.2	37.5

**Table 3:** Agreement between results obtained with chemical spot test and FT-IR in the pure stone group.

Pure stones			
Substances detected (*n*)	Agreement (*n*)	Partial agreement (*n*)	Disagreement (*n*)
Calcium oxalate monohydrate (14)	10	3	1
Anhydrous uric acid (6)	0	0	6
Cystine (2)	1	1	0
Atazanavir (1)	0	0	1

Total (23)	11 (47.8%)	4 (17.4%)	8 (34.8%)

**Table 4:** Agreement between results obtained with chemical spot test and FT-IR in the pure stone with substances in trace group.

Pure stones with substance in trace			
Components detected (*n*)	Agreement (*n*)	Partial agreement (*n*)	Disagreement (*n*)
Calcium oxalate monohydrate + carbonate apatite trace (4)	0	3	1
Calcium oxalate dihydrate + carbonate apatite trace (1)	0	1	0
Calcium oxalate monohydrate + calcium oxalate dihydrate trace (1)	0	1	0

Total (6)	0 (0.0%)	5 (83.3%)	1 (16.7%)

**Table 5:** Agreement between results obtained with chemical spot test and FT-IR in the mixed stone group.

Mixed stones			
Substances detected (*n*)	Agreement (*n*)	Partial agreement (*n*)	Disagreement (*n*)
Anhydrous uric acid + calcium oxalate monohydrate (3)	2	0	1
Calcium oxalate monohydrate + calcium oxalate dihydrate + carbonate apatite (4)	0	2	2
Magnesium ammonium phosphate + carbonate apatite + calcium oxalate monohydrate (1)	0	0	1
Carbonate apatite + magnesium ammonium phosphate + protein (1)	0	0	1
Anhydrous uric acid + calcium oxalate monohydrate + carbonate apatite (1)	0	0	1
Anhydrous uric acid + dihydrate uric acid (2)	1	0	1
Carbonate apatite + calcium oxalate monohydrate + calcium oxalate dihydrate (3)	0	0	3
Calcium oxalate monohydrate + carbonate apatite (2)	0	1	1
Calcium oxalate monohydrate + calcium oxalate dihydrate (1)	0	1	0
Calcium oxalate dihydrate + carbonate apatite + protein (1)	0	0	1

Total (19)	3 (15.8%)	4 (21.0%)	12 (63.2%)

## Abbreviations

COM: Calcium oxalate monohydrate
COD: Calcium oxalate dihydrate
CA: Carbonate apatite
PAM: Magnesium ammonium phosphate
AU0: Uric acid anhydrous
AU2: Uric acid dihydrate
FT-IR: Fourier transform infrared spectroscopy.
